# Supplementary figures and images for: Proteomic identification of biomarkers in maternal plasma that predict the outcome of rescue cerclage for cervical insufficiency
Source: PLoS One. 2021 Apr 15;16(4):e0250031. doi: 10.1371/journal.pone.0250031 (PMC8049309; doi:10.1371/journal.pone.0250031)

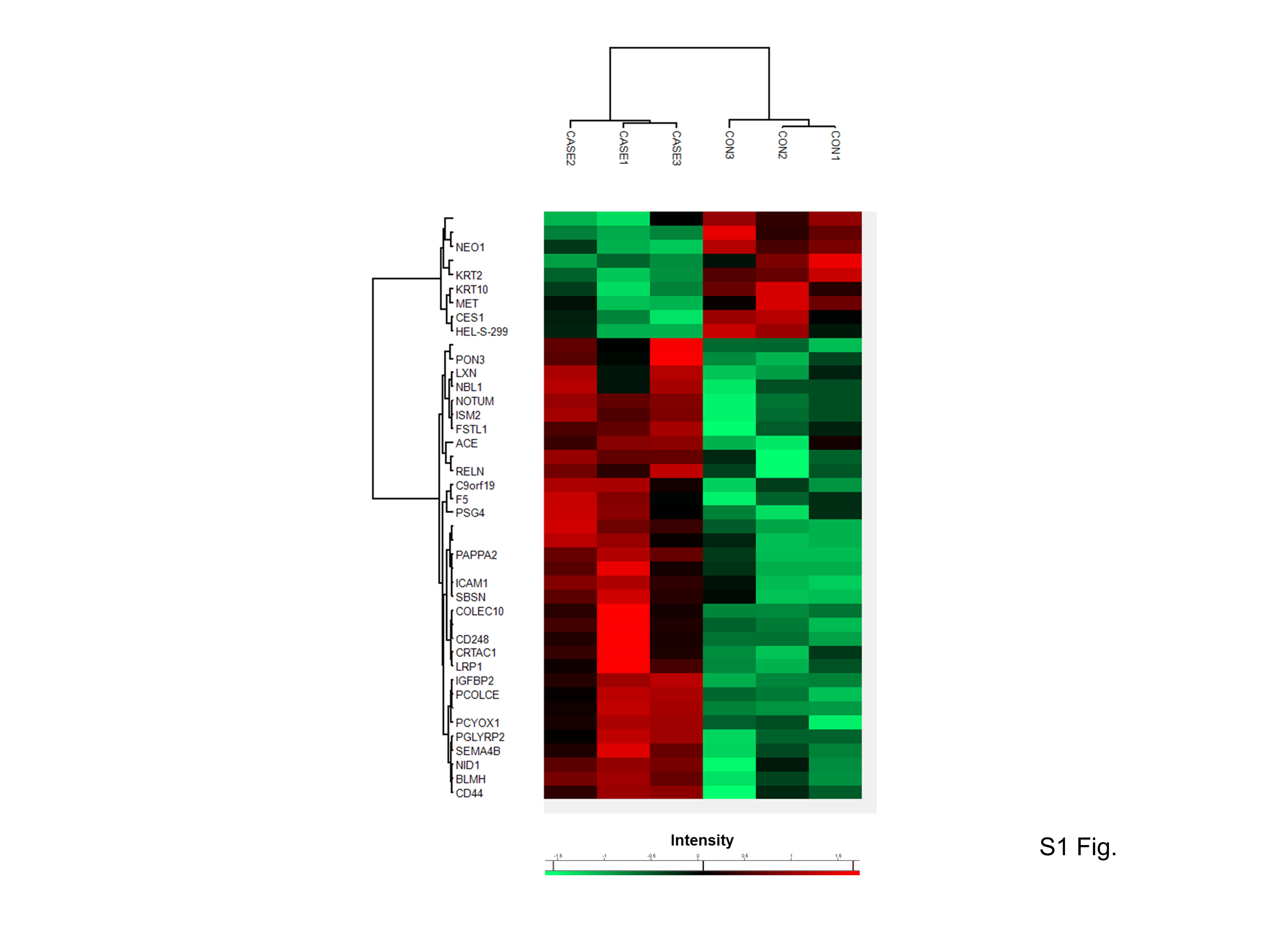

Supplement: S1 Fig — Case and control refer to plasma samples acquired from patients who had subsequent spontaneous preterm delivery at <33 weeks after cerclage placement (case) and who delivered at ≥33 weeks (control). (red = increased, green = decreased). (TIF) [file pone.0250031.s006.tif]

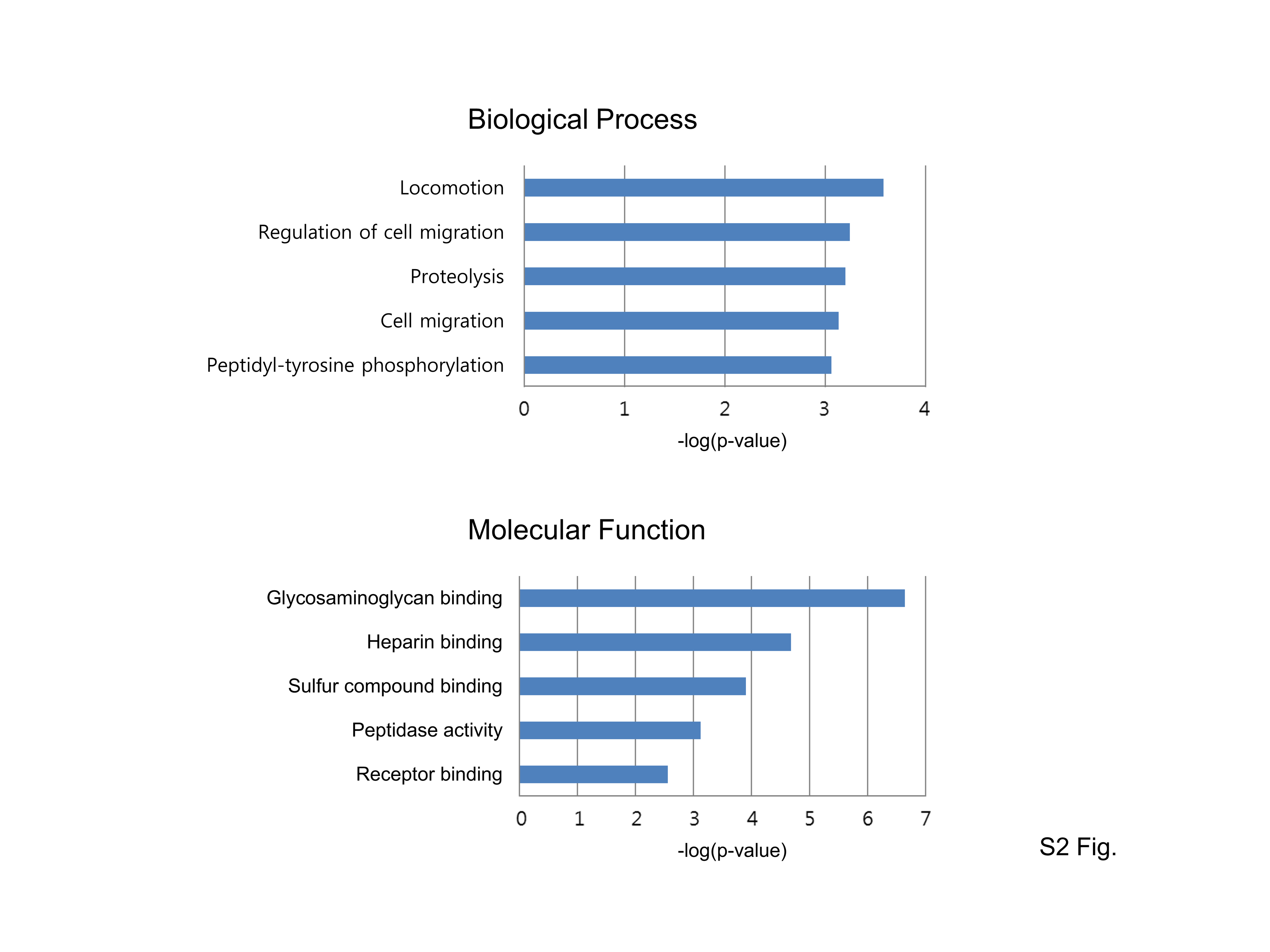

Supplement: S2 Fig — (TIF) [file pone.0250031.s007.tif]

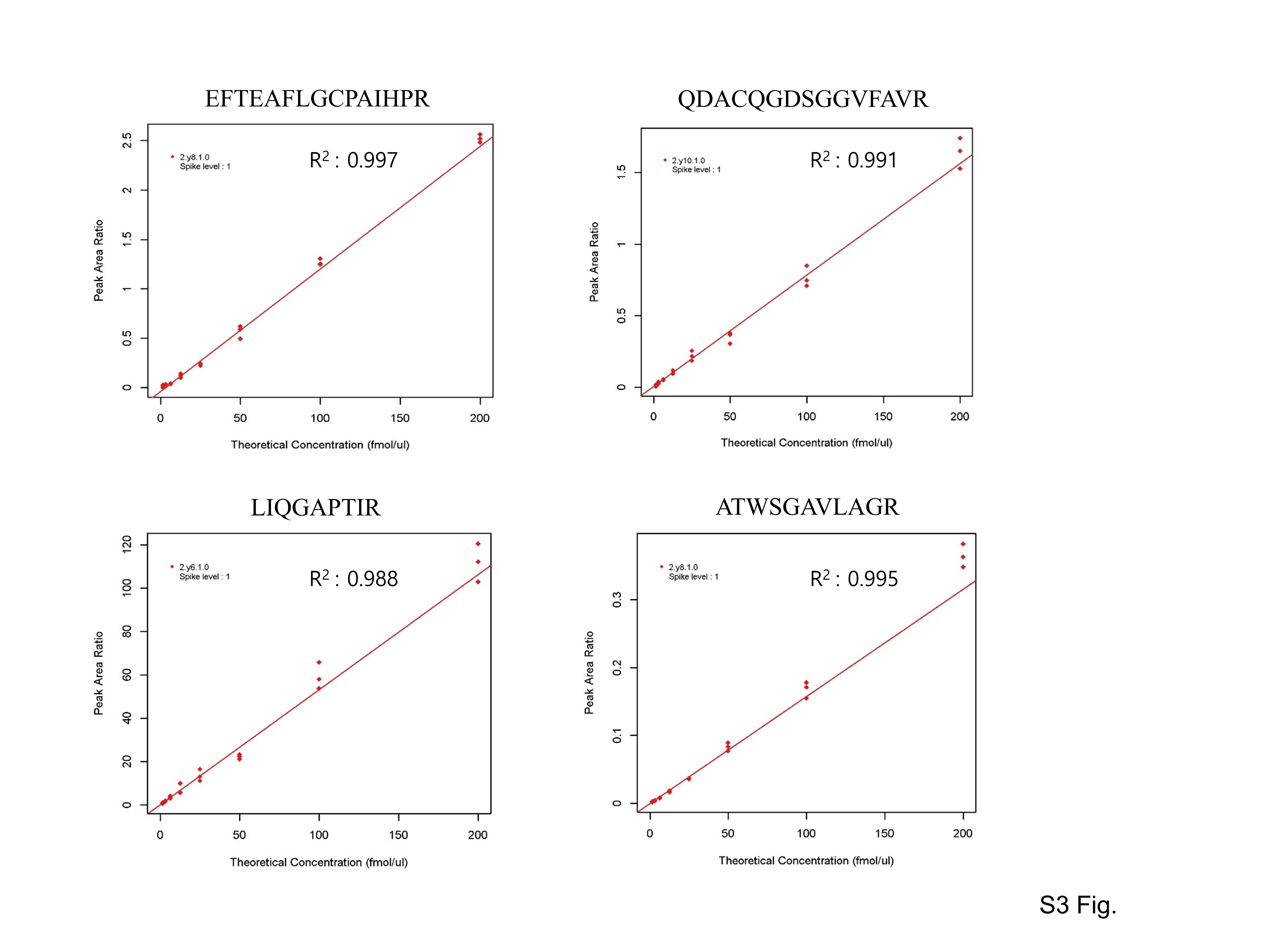

Supplement: S3 Fig — (TIF) [file pone.0250031.s008.tif]
