# Supplementary material for: Proteomic identification of biomarkers in maternal plasma that predict the outcome of rescue cerclage for cervical insufficiency
Source: PLoS One. 2021 Apr 15;16(4):e0250031. doi: 10.1371/journal.pone.0250031 (PMC8049309; doi:10.1371/journal.pone.0250031)
Supplement: S3 File — (PDF) [file pone.0250031.s011.pdf]

- **Supplementary Materials** -

Immunoaffinity depletion of high-abundance proteins and in-solution tryptic digestion

Protein concentrations were determined using a bicinchoninic acid assay (Micro BCA Protein Assay Kit, Thermo Fisher Scientific, Bremen, Germany) in each of 20 discovery cohort samples consisting of control (n = 10) and case (n = 10) groups. In each group, sets of three, three, and four plasma samples were pooled with equal amounts per sample, resulting in three sets of pooled samples for control and case groups. The pooled plasma samples were then depleted of the 14 most abundant proteins (albumin, alpha-1 acid glycoprotein, alpha-1 antitrypsin, alpha-2 macroglobulin, apolipoprotein A-I, apolipoprotein A-II, complement C3, fibrinogen, haptoglobin, immunoglobulin A, immunoglobulin G, immunoglobulin M, serotransferrin, and transthyretin) using a multiple affinity removal system, with a MARS-14 column (4.6 × 100 mm; Agilent, Santa Clara, CA, USA), according the manufacturer's instructions, with some modifications. An Infinity 1260 Bioinert HPLC system (Agilent) with an automated fraction collector was used to automatically collect flow-through fractions. Depleted plasma samples were concentrated by centrifugal filtration using a 3 kDa Amicon® filter (Milipore, Burlington, MA, USA). After the proteins were reduced and alkylated with dithiothreitol (DTT) and iodoacetamide (IAA), respectively; protein digestion was performed at 37°C overnight with a trypsin/LysC mixture at a 100:1 protein:protease ratio [w/w].

Tandem mass tag labeling

Six-plex tandem mass tag (TMT) labeling was performed according to the manufacturer's

instructions, with some modifications. Briefly, the concentration of peptides obtained by tryptic digestion was measured by tryptophan assay. TMT reagents (0.8 mg) were dissolved in anhydrous acetonitrile (ACN) and 10 µL of the resulting solution was added to the peptides (50 µg) in each set of pooled plasma samples, along with ACN, to achieve a final ACN concentration of 30% (v/v). The three control sample sets were labeled with 126, 127, and 128, while the three case sample sets were labeled with 129, 130, and 131 tags. For normalization and confirmation of labeling quality, 500 ng of peptides derived from the ovalbumin standard protein were spiked into each of the TMT-labeled samples. Following incubation at room temperature for 1 h, the labeling reaction was quenched with hydroxylamine at a final concentration of 0.3% (v/v). The TMT-labeled samples were the combined in a 1:1:1:1:1:1 ratio, dried using SpeedVac and subjected to C18 solid-phase extraction for desalting.

High pH reversed-phase peptide fractionation

The TMT-labeled peptide samples were fractionated using an Agilent 1260 Bio-inert HPLC system equipped with an analytical column (4.6 x 250 mm, 5 µm). High-pH reversed-phase liquid chromatography was performed at a flow rate of 0.8 mL/min for 60 min using a gradient of solvent A (15 mM ammonium hydroxide in water) and solvent B (15 mM ammonium hydroxide in 90% ACN). Peptides were separated with a gradient of 5% to 35% ACN at 0.2 mL/min. A total of 96 fractions was collected and non-contiguously concatenated into 24 fractions.

Liquid chromatography-electrospray ionization-tandem mass spectrometry (LC-ESI-MS/MS) analysis

The fractionated peptide samples were analyzed using a Quadrupole Orbitrap Q-Exactive Plus mass spectrometer (Thermo Fisher Scientific) coupled to an Ultimate 300 RSLC system (Dionex, Sunnyvale, CA, USA) via a nanoelectrospray source. Fractionated peptide samples were separated on a reversed-phase analytical column (Themo Scientific PepMap RSLC, 75 μm inner diameter, 50 cm length) for 240 min, with a gradient from 7% to 32% ACN at 300 nL/min. The column temperature was set to a constant temperature of 60°C using a column heater. The survey scans (350 to 1650 m/z) were acquired with a resolution of 70,000 at *m/z* 200. A top 20 method was used to select up the 20 most abundant precursor ions with an isolation window of 1.2 m/z. The selected precursor ions were subjected to higher-energy collisional dissociation fragmentation at a normalized collision energy of 32 with a resolution of 35,000 at *m/z* 200. The maximum ion injection time for the full mass spectrometry (MS) and MS/MS scans were 20 and 100 ms, respectively.

Data processing for protein identification and quantification

Raw MS files were processed using the Proteome Discoverer 2.2 software interface with the SEQUEST-HT search engine, based on the UniProt HUMAN database (88657 entries; released in December 2014), including forward and reverse protein sequences and common contaminants. Searches were performed using a precursor ion tolerance of 10 ppm and a fragment ion tolerance of 20 ppm. TMT tags on lysine residues, peptide N termini (+229.163 Da), and carbamidomethylation of cysteine residues (+57.021 Da) were set as fixed

modifications, while oxidation of methionine residues (+15.995 Da) was set as a variable modification. The false discovery rates for all peptide spectrum matches (PSMs), peptides, and protein assignments were calculated using the included Percolator software package. Reporter ion quantification was performed in the MS2 channel, with a 20-ppm mass tolerance. Only the PSMs that contained all 6 reporter ions were considered. Each reporter ion channel was summed across all quantified proteins and was normalized assuming equal protein loading of all 6 samples.

Bioinformatics analysis for selecting differentially expressed proteins

Bioinformatics analysis was performed using the Perseus software.[1] Statistical analysis was performed on logarithmized intensities of TMT-reporter ions and subsequently, a two-sample Student's *t*-test was performed to identify significantly expressed proteins with a *P* value cutoff of 0.05. Gene ontology analysis was performed using the DAVID bioinformatics tool (http://david.abcc.ncifcrif.gov/).

Plasma sample preparation for quantification of target peptides with LC-multiple reaction monitoring (MRM)-MS

Thirty microliters of each plasma sample diluted with 50 μL of buffer A (Agilent) was passed through 0.22 μm spin filters (Agilent). Six highly abundant human plasma proteins were then removed using a MARS-6 column (Agilent). An Infinity 1260 Bioinert HPLC system with an automated fraction collector was used to automatically collect flow-through fractions.

Depleted plasma was concentrated by centrifugal filtration using a 3 kDa molecular weight cut-off filter (Amicon® Ultra 3K, Millipore). The concentrated plasma protein was quantified by Bradford assay (Quick Start Bradford Protein Assay kit; BioRad, Hercules, CA, USA). Equal amounts of protein (100 μg) from each sample were denatured with 0.1% (final concentration) RapiGest™ surfactant (Waters, Milford, CT, USA) for 20 min at 80°C. Denatured proteins were reduced by the addition of 50 mM DTT (Merck, Darmstadt, Germany) in 50 mM ammonium bicarbonate, followed by incubation at 60°C for 30 min. The proteins were then alkylated by the addition of 150 mM IAA (Sigma, St. Louis, MO, USA), followed by incubation at room temperature for 30 min in the dark. Sequencing-grade modified trypsin was added to samples at a 50:1 ratio (plasma proteins substrate:trypsin ratio [w/w]). Trypsin digestion was performed for 16 h at 37°C. Tryptic digestion was quenched by the addition of 1% trifluoroacetic at a final concentration of 0.5 % (v/v), followed by incubation for 45 min at 37°C. The peptides generated from trypsin digestion were desalted using OASIS HLB 1-cc (30 mg) desalting cartridges (Waters). The desalted peptides were lyophilized on a speed vacuum centrifuge (CcntriVap® centrifugal concentrator, LABCONCO, Kansas City, MO, USA) and stored at -80°C until use. Prior to LC-MRM-MS analysis, the samples were reconstituted in 0.1% formic acid in 1% ACN and mixed with mixtures of stable heavy isotope-labeled peptides. The concentrations of spiked heavy peptides were adjusted to the peak areas of the corresponding endogenous peptides so that the light (endogenous peptide) to heavy peptide peak area ratios ranged from 0.1 to 10.

LC-MRM-MS analysis

MRM analysis was performed on a 6495 Triple-Quadrupole Mass Spectrometer coupled to an Agilent 1260 Infinity HPLC system (Agilent). Twenty micrograms of tryptic peptides from each plasma sample was loaded onto a reversed-phase analytical column (Agilent ZORBAX SB-C18, 3.5 μm, 15 cm in length, 500 μm inner diameter). The temperature of the analytical column was maintained at 40°C. Peptide separation was performed at a flow rate of 18 μL/min on a linear gradient of mobile phase B (0.1% formic acid in ACN) from 2 % to 40 % over 45 min. The total run time was 73 min (mobile phase A: 0.1% formic acid in water, mobile phase B: 0.1% formic acid in ACN). A triple quadrupole LC/MS system (6495 LCMS, Agilent) was used for the detection of m/z and the signal intensity of peptides eluted from the analytical column. Automated optimization of the electrospray source parameters and mass axis calibration in ESI positive mode were performed with tuning solution (G1969-85000, Agilent), using the autotune function in MassHunter Workstation software (ver B.08.00, Agilent). The drying gas temperature was set at 250°C, at a flow rate of 15 L/min, while the sheath gas temperature was set at 300°C, at a flow rate of 12 L/min. The nebulizer gas flow was set at 30 psi. MRM-MS analysis was performed in the positive ion mode, with the ion spray capillary voltage and nozzle voltage set at 3,000 and 1,200 V, respectively. The delta electron multiplier voltage was set at 300 V and the cell accelerator voltage and fragment voltages were set at 5 V and 380 V, respectively. The resolution of MS quadrupole-1 and quadrupole-3 was set to unit resolution (0.7 FWHM).

MRM-MS method optimization and data processing for target protein verification

Target peptides and transition lists for MRM method development were generated from 40

DEPs extracted from TMT-based proteome profiling results, using Skyline software (ver 3.6; University of Washington, Seattle, WA, USA). To evaluate the suitability of the heavy peptides for LC-MRM-MS method optimization, we identified the retention time, optimized collisional energy values, and MS signal intensity for heavy peptides by spiking them into a tryptic peptide matrix obtained from the pooled plasma samples used for discovery experiments. All raw MRM-MS data were processed using Skyline software. All integrated peaks were manually inspected to confirm correct peak detection and accurate integration. The MRM acquisition method was initially built with eight to fourteen precursor/fragment ion pairs (transitions) for each light and heavy peptide. Each light and heavy transition was carefully reviewed to refine the transitions showing a better MS response, no interference, and consistency of signal intensity over consecutive LC-MS runs. Peptides meeting the following criteria were excluded during the optimization process: 1) low light (endogenous) and heavy MS response, 2) sub-optimal chromatographic characteristics (severe peak tailing), and 3) failure to build up two or more transitions that were reproducibly detected and free of interference in the plasma matrix. Reverse response curves for examining linearity were generated from MRM-MS data from each serially diluted heavy peptide spiked into 25 μg of trypsin-digested plasma matrix. Eight concentration points (approximately 1.56–200 fmol/μL) were used and a trypsin-digested pooled plasma matrix, without heavy peptides added, was used as a blank to estimate background levels. Triplicate MRM-MS runs were performed at each concentration point. Plots of the peak area ratio of heavy to light peptides versus the heavy peptide area of eight concentration points were generated and linearity and coefficients of variation (CVs) were evaluated using QUASAR software. MRM transitions of each heavy peptide with a CV $< 20\%$ and a coefficient of determination > 0.98 were selected as refined

transitions for quantification of the corresponding endogenous peptides. The target cycle time for the multiplexed MRM-MS method was set at 1,500 ms. Minimum/maximum dwell times were adjusted automatically, depending on the number of concurrent transitions, using MassHunter Workstation software. The retention time tolerance window for scheduled MRM-MS methods was 2.8 min.

- Supplementary Results -

Development of the multiplexed multiple reaction monitoring (MRM) method for the quantification of target proteins using heavy peptides

From the discovery experiment, 40 differentially expressed proteins (DEPs) were found to have statistically significant changes ($P < 0.05$) between the control and case groups. FASTA-formatted amino acid sequences of the 40 DEPs were imported and lists of target surrogate peptides that met the following conditions were created using Skyline software: 1) in silico tryptic (KR|P) digestion; 2) restricting length of tryptic peptides ranging from 7 to 22 amino acids, assuming no miscleavage; and 3) peptides containing methionine residues or possible glycosylation sites (NXT/NXS and RP/KP) were excluded. Monitoring of doubly or triply charged precursor ions, singly charged fragment ions, and both b and y fragment type ions, were used for MRM transition filtering.

A total of 274 surrogate peptides was generated from the 40 DEPs. The appropriate surrogate peptides for MRM method optimization were then selected according to the following criteria: 1) peptides identified from discovery experiments or present in a plasma proteome MS/MS library constructed in-house; 2) high confidence peptides (mostly higher MS and MS/MS signal intensity); and 3) peptides present in the SRM database (http://www.srmatlas.org) or PeptideAtlas (www.peptideatlas.org). Peptides meeting at least one criterion were selected. Finally, 82 surrogate peptides, with mostly 2 peptides per protein (Table S4) and 82 heavy isotope-labeled peptide standards (SpikeTides^{TM}; JPT Peptide Technologies, Berlin, Germany), at an unknown purity grade, were synthesized and evaluated

for suitability. During the MRM method optimization process, 23 peptides that did not meet the criteria for MRM experiments (Supplementary Materials) were excluded. We selected 2-4 MRM transitions with the best intensity and linearity for each surrogate peptide, refining the MRM transition process. Representative response curves of the heavy peptides are shown in Figure S3. Finally, we established a multiplexed MRM method that could quantify 59 surrogate peptides derived from 37 proteins in one LC-MS injection. The optimized MRM parameters for 404 transitions for 59 peptides are shown in Table S5.

Supplementary References

1 Tyanova S, Temu T, Sinitcyn P, Carlson A, Hein MY, Geiger T, et al. The Perseus computational platform for comprehensive analysis of (prote)omics data. *Nat Methods* 2016;13: 731-740.
